# Supplementary material for: Metallic glass-based triboelectric nanogenerators
Source: Nat Commun. 2023 Feb 23;14:1023. doi: 10.1038/s41467-023-36675-x (PMC9950355; doi:10.1038/s41467-023-36675-x)
Supplement: Supplementary file 1 — Supplementary Information [file 41467_2023_36675_MOESM1_ESM.pdf]

Supplementary Information

Metallic glass-based triboelectric nanogenerators

Xin Xia[1,2 #], Ziqing Zhou[3, #], Yinghui Shang[3,4], Yong Yang[3,5,6,*], Yunlong Zi[1,2,7,8,*]

[1]Department of Mechanical and Automation Engineering, The Chinese University of Hong Kong, Shatin, N.T., Hong Kong SAR, China

[2] Thrust of Sustainable Energy and Environment, The Hong Kong University of Science and Technology (Guangzhou), Nansha, Guangzhou, Guangdong 511400, China

[3] Department of Mechanical Engineering, College of Engineering, City University of Hong Kong, Kowloon Tong, Kowloon, Hong Kong, China

[4] City University of Hong Kong (Dongguan), Dongguan 523000, China

[5.]Department of Materials Science and Engineering, College of Engineering, City University of Hong Kong, Kowloon Tong, Kowloon, Hong Kong, China

[6.] Department of Advanced Design and System Engineering, College of Engineering, City University of Hong Kong, Kowloon Tong, Kowloon, Hong Kong, China

[7] HKUST Shenzhen-Hong Kong Collaborative Innovation Research Institute, Futian, Shenzhen, Guangdong, China

[8] Department of Mechanical and Aerospace Engineering, The Hong Kong University of Science and Technology, Clear Water Bay, Hong Kong SAR, China

[*] *Corresponding author: yunlongzi@gmail.com (Y. Z.); yonyang@cityu.edu.hk (Y. Y.)*

#. The authors contributed equally to this work.

Contents of supplementary information:

Supplementary Figures

Supplementary Figure 1. Confirmation of amorphous phase. XRD, diffraction image and high-resolution TEM image.

Supplementary Figure 2. SEM-EDX results for atom distributions.

Supplementary Figure 3. SEM images of the top surface of $Zr_{45}Cu_{35}Al_{20}$ with different scales.

Supplementary Figure 4. SEM images of the top surface of $Zr_{45}Cu_{45}Al_{15}$ with different scales.

Supplementary Figure 5. SEM images of the top surface of $Zr_{50}Cu_{40}Al_{10}$ with different scales.

Supplementary Figure 6. SEM images for Cu plate. Obvious scratches existed on the Cu surface.

Supplementary Figure 7. SEM images for the bottom surfaces of $Zr_{45}Cu_{40}Al_{15}$

Supplementary Figure 8. SEM images across the thickness of different samples.

Supplementary Figure 9. AFM images for surface roughness. The scanning size for each sample are 8μm × 8μm.

Supplementary Figure 10. Analysis of wear resistance.

Supplementary Figure 11. Photographs of *Drainage-method* for atomic density.

Supplementary Figure 12. Dynamic friction coefficient measurement of different sample surfaces.

Supplementary Figure 13. SEM images of the scratches on surface after indentation measurement.

Supplementary Figure 14. Photograph of the platform setup.

Supplementary Figure 15. Output performance of (a) $Zr_{50}Cu_{40}Al_{10}$ and (b) $Zr_{45}Cu_{35}Al_{20}$ with soft substrate contacting with FEP.

Supplementary Figure 16. V_{OC} and Q_{SC} of different samples with soft substrate.

Supplementary Figure 17. V_{OC} and Q_{SC} of different samples with hard substrate.

Supplementary Figure 18. Performance evaluation of LS mode TENG.

Supplementary Figure 19. Photograph to illustrate the height controlled by the lifting elevator.

Supplementary Figure 20. V_{OC} and Q_{SC} of different MG/polymer pairs.

Supplementary Figure 21. V_{OC} and Q_{SC} of different Cu/polymer pairs.

Supplementary Figure 22. V_{OC} and Q_{SC} of MG or Cu served as the induction metals.

Supplementary Figure 23. V_{OC} and Q_{SC} of MG or Cu under different RH.

Supplementary Figure 24 Schematic diagrams of charge transfer mechanism of MG and MG with hydrogen bonding (MG—H-O-H) contacting with FEP.

Supplementary Figure 25. Durability test.

Supplementary Figure 26. Surface morphologies change before and after long-term durability test。

Supplementary Figure 27. Schematic structure of T-TENG.

Supplementary Figure 28. System configuration for powering commercial LEDs.

Supplementary Figure 29. Circuit for measuring the high frequency current.

Supplementary Figure 30. Mechanical illustrations.

Supplementary Figure 31. Resistance impedance of T-TENG. Relative humidity around 68%.

Supplementary Tables

Supplementary Table 1. Atom compositions of different samples from SEM-EDX results.

Supplementary Table 2. Mechanical properties of different samples.

Supplementary Table 3. Atomic density of different samples.

Supplementary Table 4. Friction coefficient (dynamic) of different samples.

Supplementary Table 5. Durability test of different samples.

Supplementary Table 6. Elastic strain limit of different MG samples.

Supplementary Notes

Supplementary Note 1. Calculation of the deformation involved in different devices.

Supplementary Note 2. Discussion of the different power output curve by T-TENG.

Supplementary Movies

Supplementary Movie 1. 200 LEDs connected in series powered by the T-TENG.

Supplementary Movie 2. LED of 3W powered by the T-TENG.

Supplementary Movie 3. 3 3W-LEDs (9W in total) connected in series powered by the T-TENG in dark room.

Supplementary Movie 4. 3 3W-LEDs (9W in total) connected in series powered by the T-TENG in bright room.

Supplementary Movie 5. Demonstration of the small vertical load during measurement.

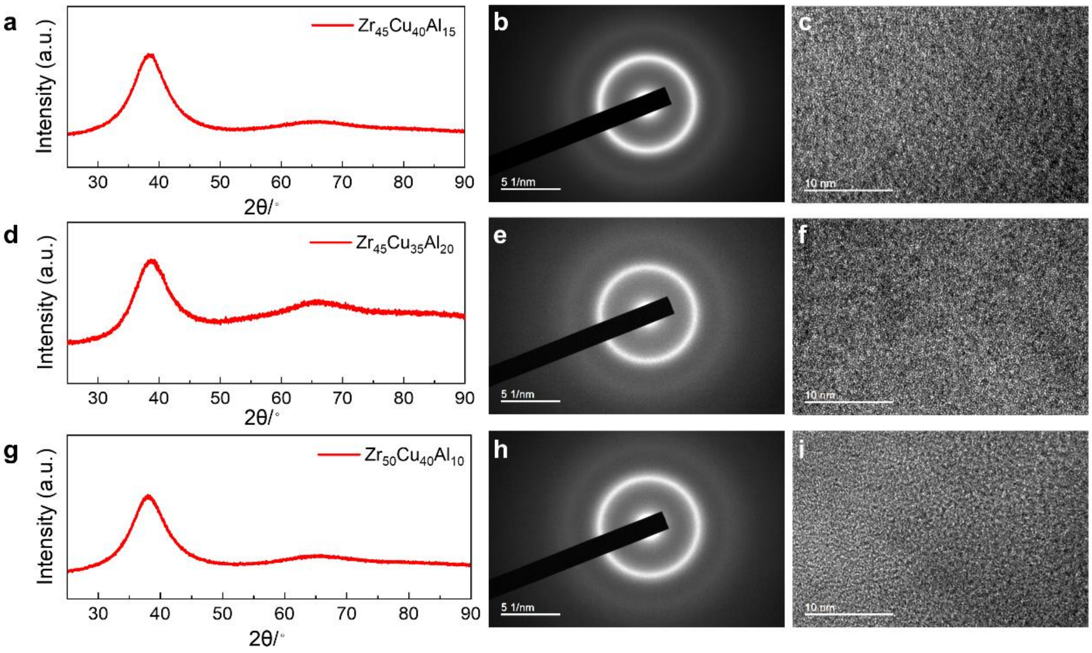

Supplementary Figure 1. Confirmation of amorphous phase. XRD, diffraction image and high-resolution TEM image of (a-c) $Zr_{45}Cu_{40}Al_{15}$, (d-f) $Zr_{45}Cu_{35}Al_{20}$ and (g-i) $Zr_{50}Cu_{40}Al_{10}$.

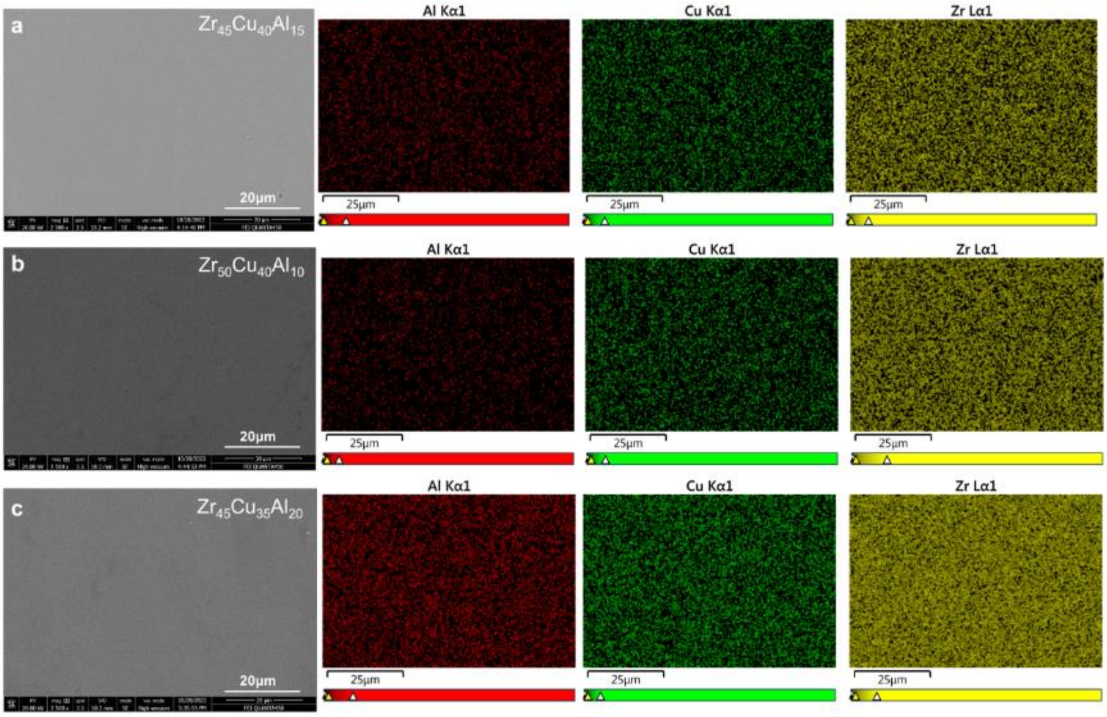

Supplementary Figure 2. SEM-EDX results for atom distributions (a) $Zr_{45}Cu_{40}Al_{15}$; (b) $Zr_{50}Cu_{40}Al_{10}$; (c) $Zr_{45}Cu_{35}Al_{20}$.

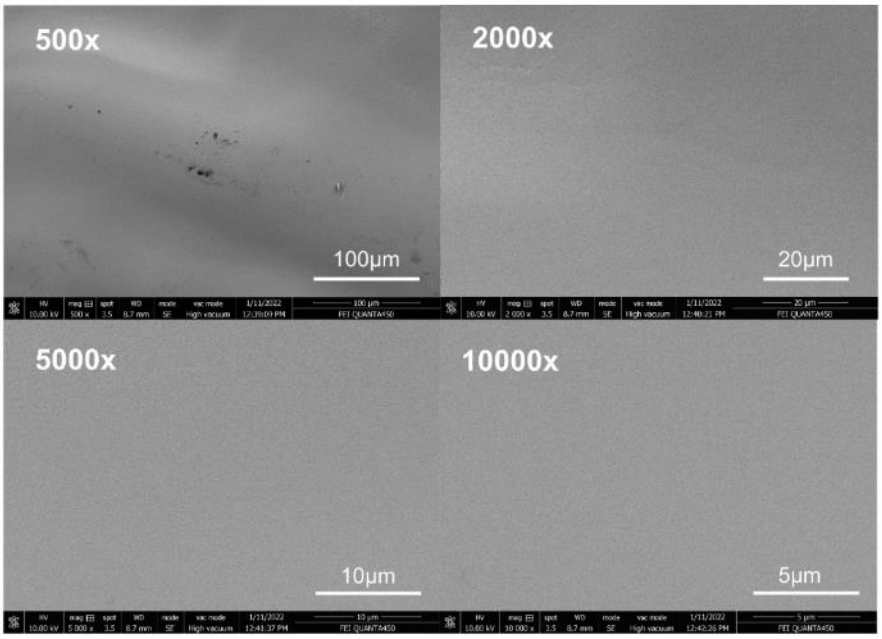

Supplementary Figure 3. SEM images of the top surface of $Zr_{45}Cu_{35}Al_{20}$ with different scales.

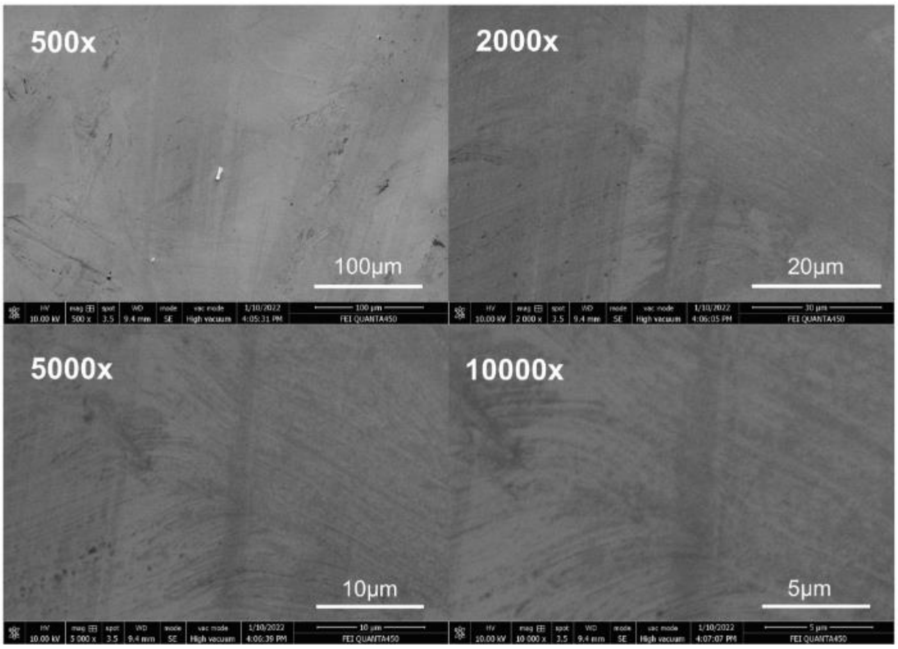

Supplementary Figure 4. SEM images of the top surface of $Zr_{45}Cu_{45}Al_{15}$ with different scales.

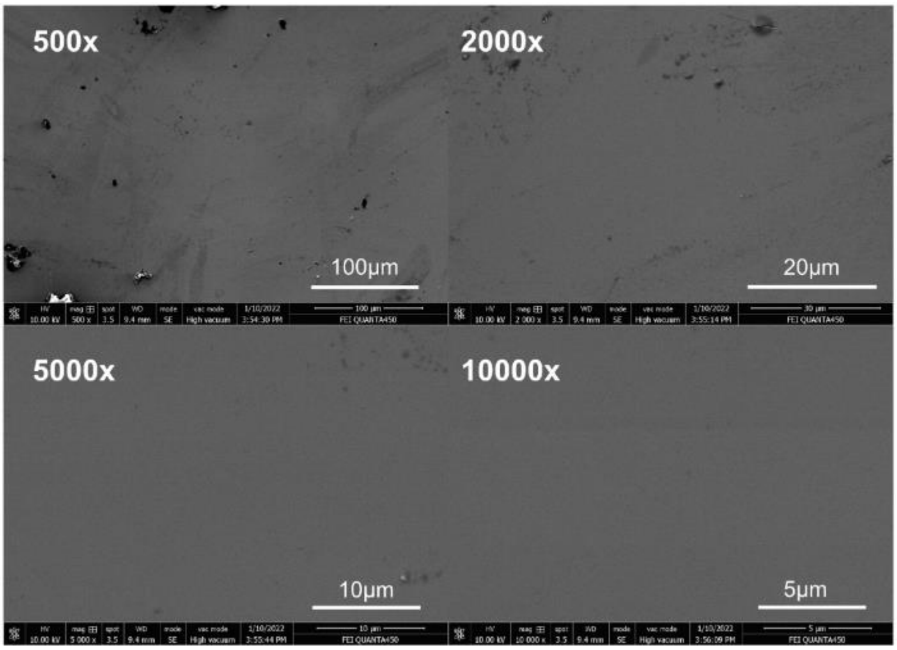

Supplementary Figure 5. SEM images of the top surface of $Zr_{50}Cu_{40}Al_{10}$ with different scales.

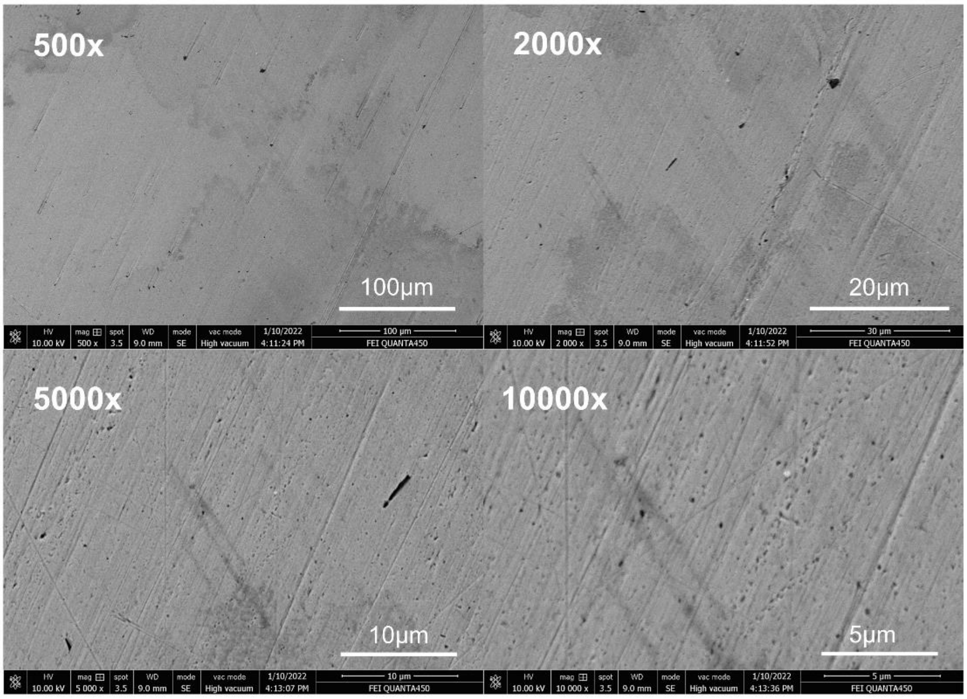

Supplementary Figure 6. SEM images for Cu plate. Obvious scratches existed on the Cu surface.

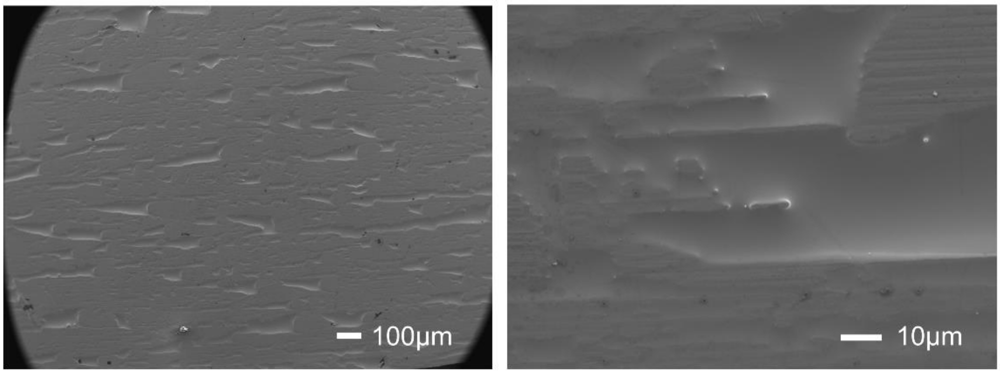

Supplementary Figure 7. SEM images for the bottom surfaces of $Zr_{45}Cu_{40}Al_{15}$

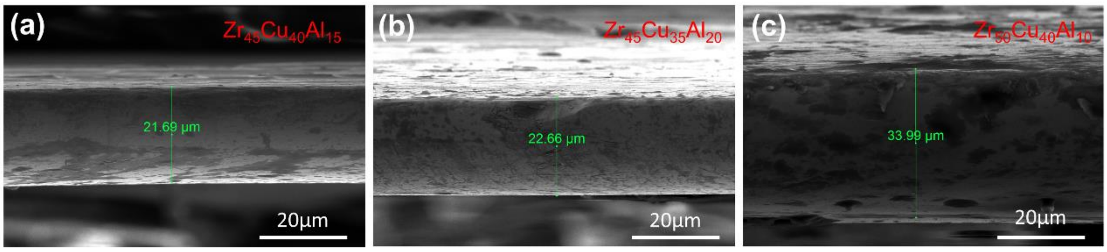

Supplementary Figure 8. SEM images across the thickness of different samples. (a) $Zr_{45}Cu_{40}Al_{15}$; (b) $Zr_{45}Cu_{35}Al_{20}$; (c) $Zr_{50}Cu_{40}Al_{10}$.

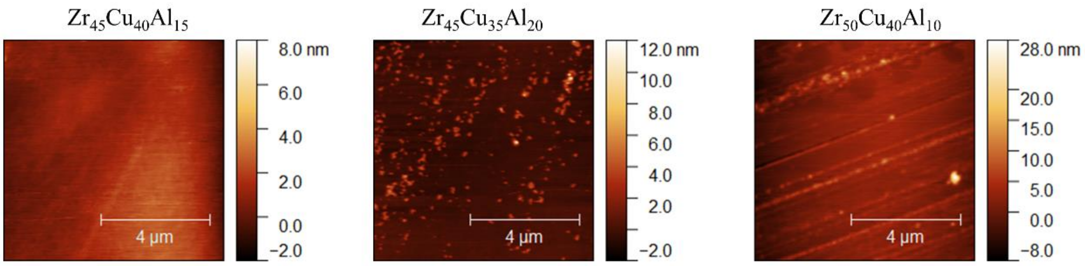

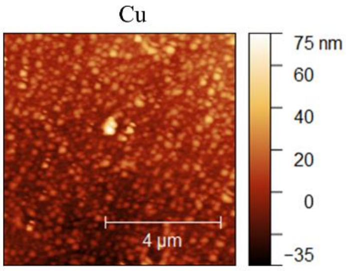

**Samples**	**$Zr_{45}Cu_{40}Al_{15}$**	**$Zr_{45}Cu_{35}Al_{20}$**	**$Zr_{50}Cu_{40}Al_{10}$**	**Cu**
Mean Roughness (nm)	0.727	0.441	2.452	12.44
RMS Roughness (nm)	0.597	0.761	1.587	9.85

Supplementary Figure 9. AFM images for surface roughness. The scanning size for each sample are 8μm × 8μm.

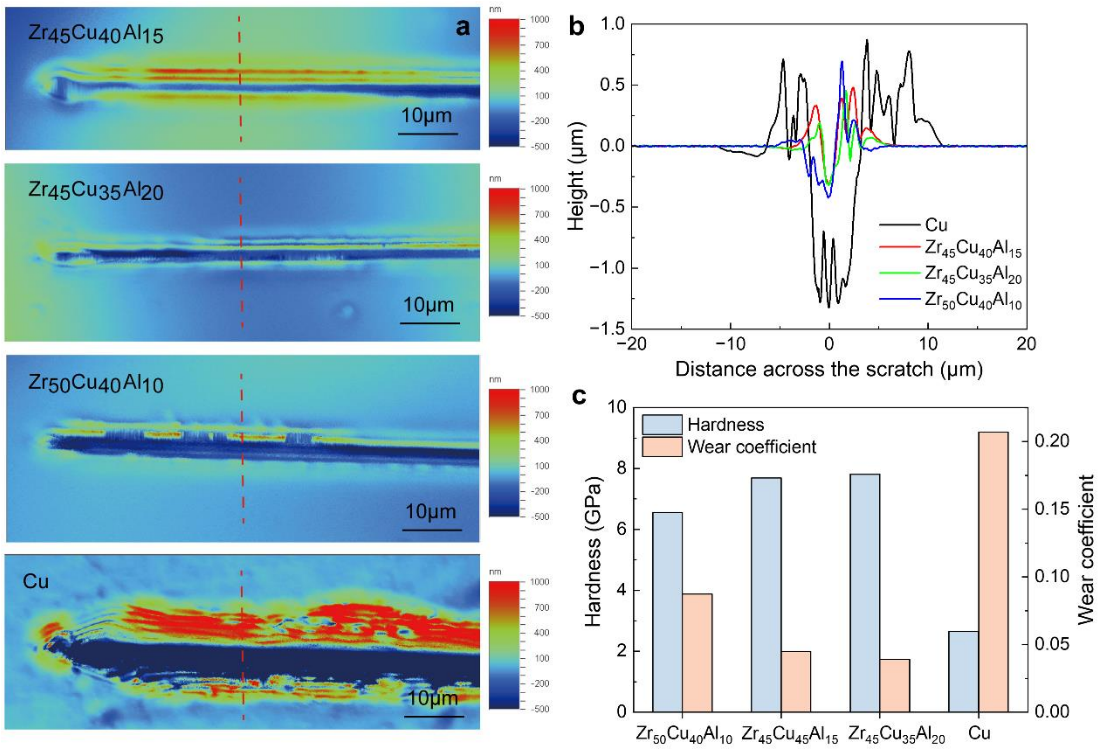

Supplementary Figure 10. Analysis of wear resistance. (a) Optical surface profiler images of different samples. (b) Profile images of scratch. (c) Mechanical properties of different samples.

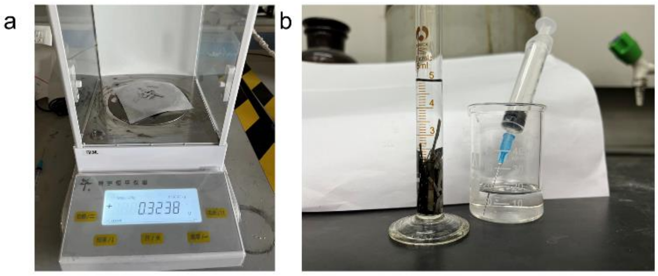

Supplementary Figure 11. Photographs of *Drainage-method* for atomic density.

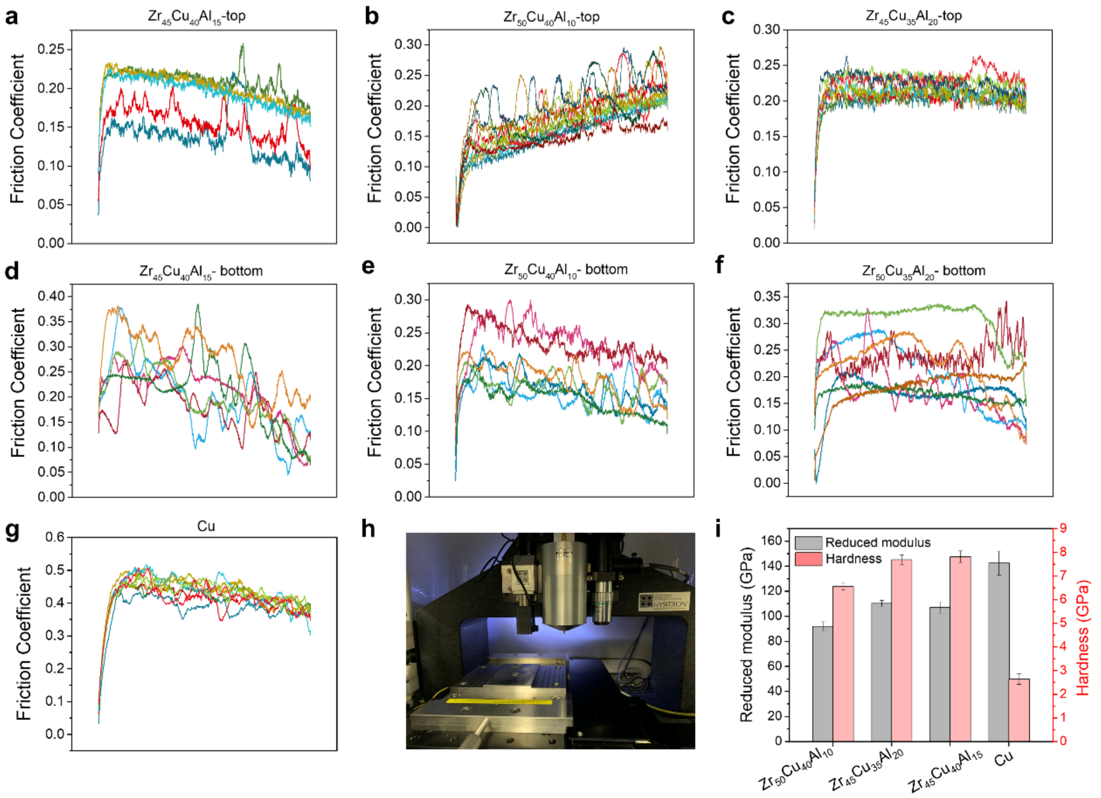

Supplementary Figure 12. Dynamic friction coefficient measurement of different sample surfaces. Results for top surfaces of (a) $Zr_{45}Cu_{40}Al_{15}$, (b)$Zr_{50}Cu_{40}Al_{10}$ and (c)$Zr_{45}Cu_{35}Al_{20}$. Results for bottom surfaces of (d) $Zr_{45}Cu_{40}Al_{15}$, (e)$Zr_{50}Cu_{40}Al_{10}$ and (f)$Zr_{45}Cu_{35}Al_{20}$. (g) Results of Cu plate. (h) photograph of the measurement system. (i) Reduced modulus and surface hardness of different samples.

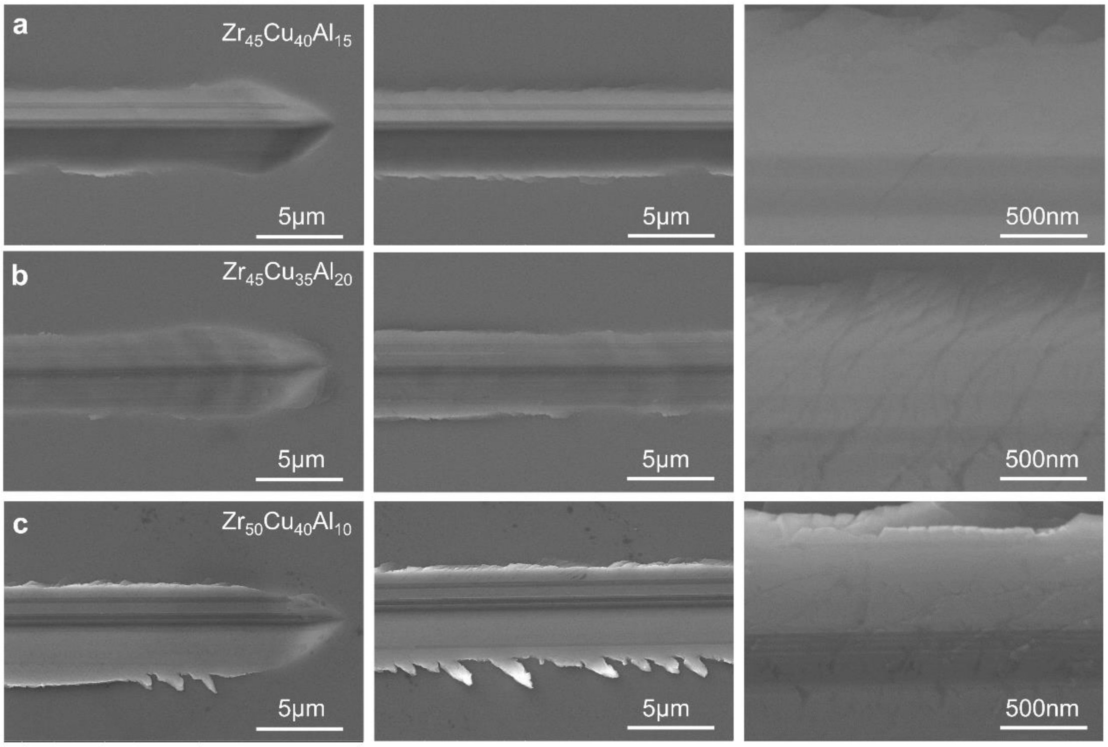

Supplementary Figure 13. SEM images of the scratches on surface after indentation measurement. (a) $Zr_{45}Cu_{40}Al_{15}$; (b) $Zr_{45}Cu_{35}Al_{20}$; (c) $Zr_{50}Cu_{40}Al_{10}$.

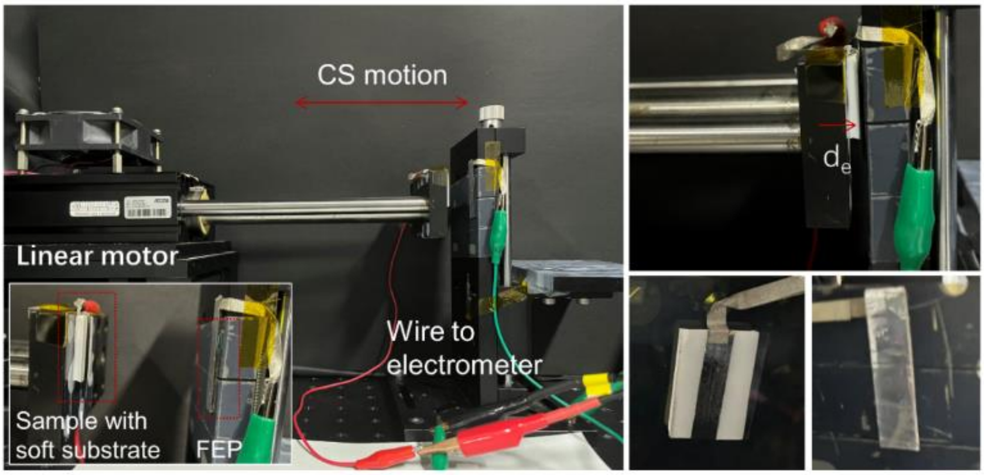

Supplementary Figure 14. Photograph of the platform setup.

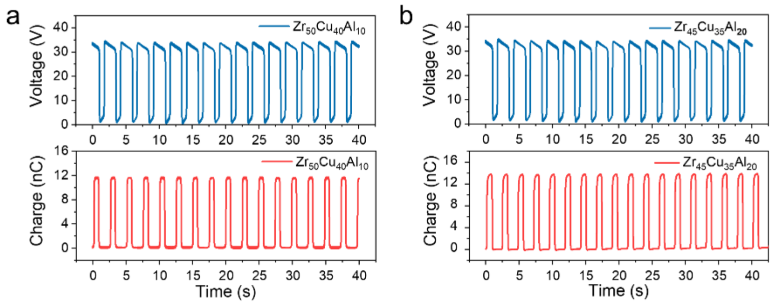

Supplementary Figure 15. Output performance of (a) $Zr_{50}Cu_{40}Al_{10}$ and (b) $Zr_{45}Cu_{35}Al_{20}$ with soft substrate contacting with FEP.

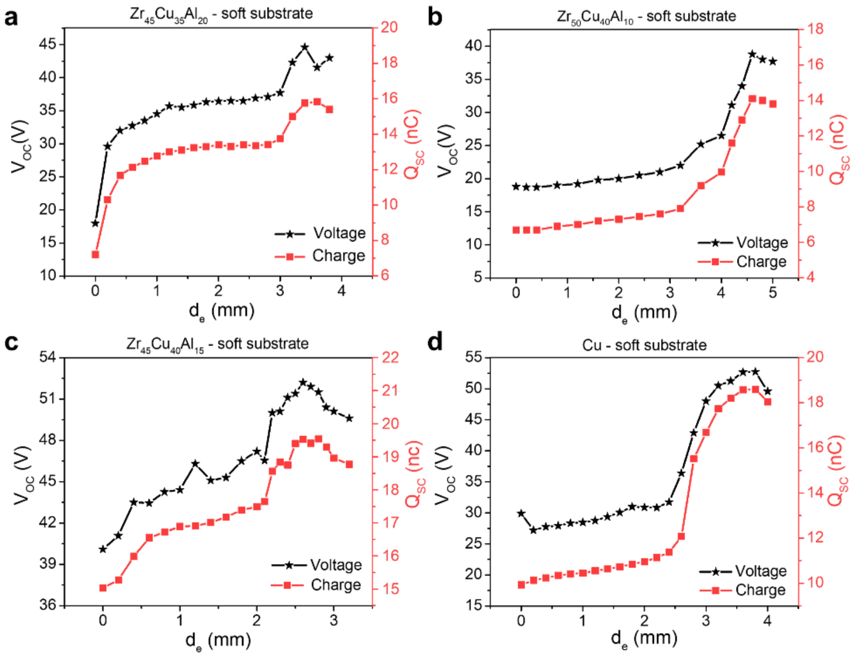

Supplementary Figure 16. V_{OC} and Q_{SC} of different samples with soft substrate. (a)$Zr_{45}Cu_{35}Al_{20}$, (b)$Zr_{50}Cu_{40}Al_{10}$, (c) $Zr_{45}Cu_{40}Al_{15}$; (d) Cu.

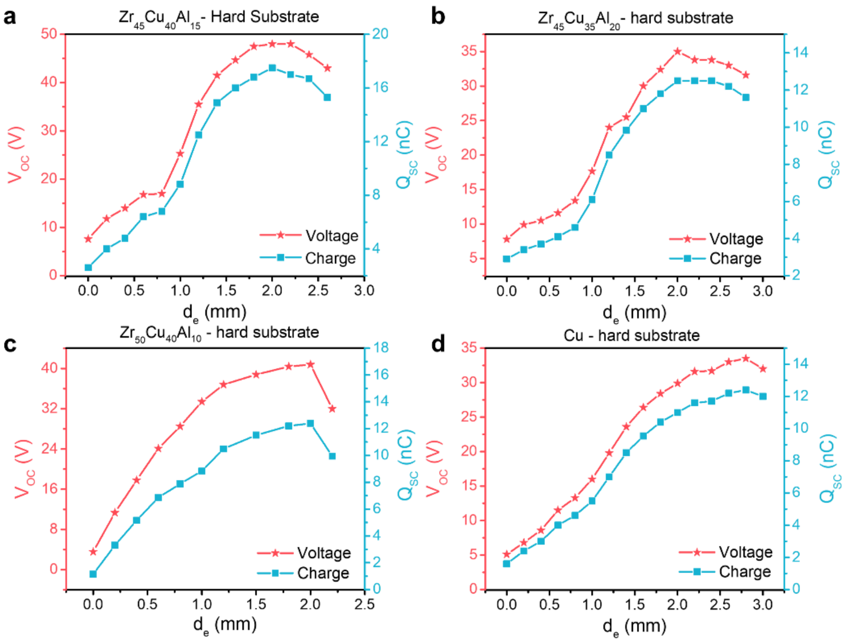

Supplementary Figure 17. V_{OC} and Q_{SC} of different samples with hard substrate. (a) $Zr_{45}Cu_{40}Al_{15}$, (b)$Zr_{45}Cu_{35}Al_{20}$, (c)$Zr_{50}Cu_{40}Al_{10}$ and (d) Cu.

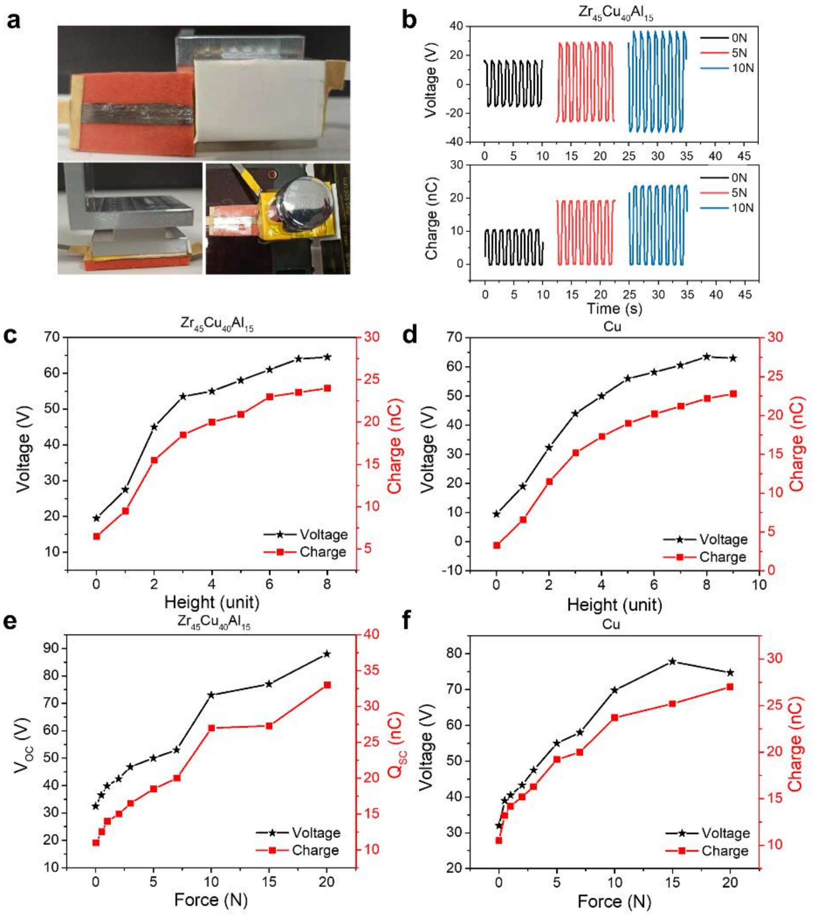

Supplementary Figure 18. Performance evaluation of LS mode TENG. (a)

Photograph of the device and experiment platform. (b) Voltage (up) and charge (bottom) output of $Zr_{45}Cu_{40}Al_{15}$/PTFE TENG with different vertical load. The voltage and charge output under different height of (c) $Zr_{45}Cu_{40}Al_{15}$ and (d) Cu. A unit was around 0.2 mm in height by the lifting elevator. The voltage and charge output under different vertical force of (e) $Zr_{45}Cu_{40}Al_{15}$ and (f) Cu.

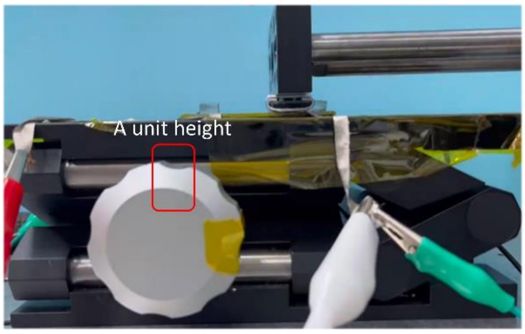

Supplementary Figure 19. Photograph to illustrate the height controlled by the lifting elevator.

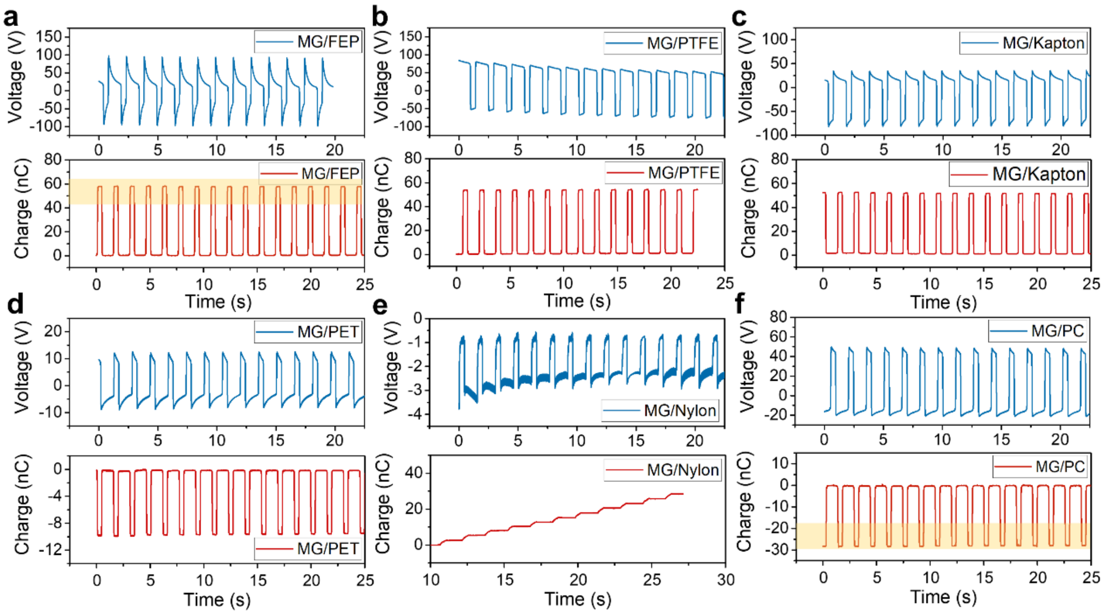

Supplementary Figure 20. V_{OC} and Q_{SC} of different MG/polymer pairs. (a) MG/FEP; (b) MG/PTFE; (c) MG/Kapton; (d) MG/PET; (e) MG/Nylon; (f) MG/PC.

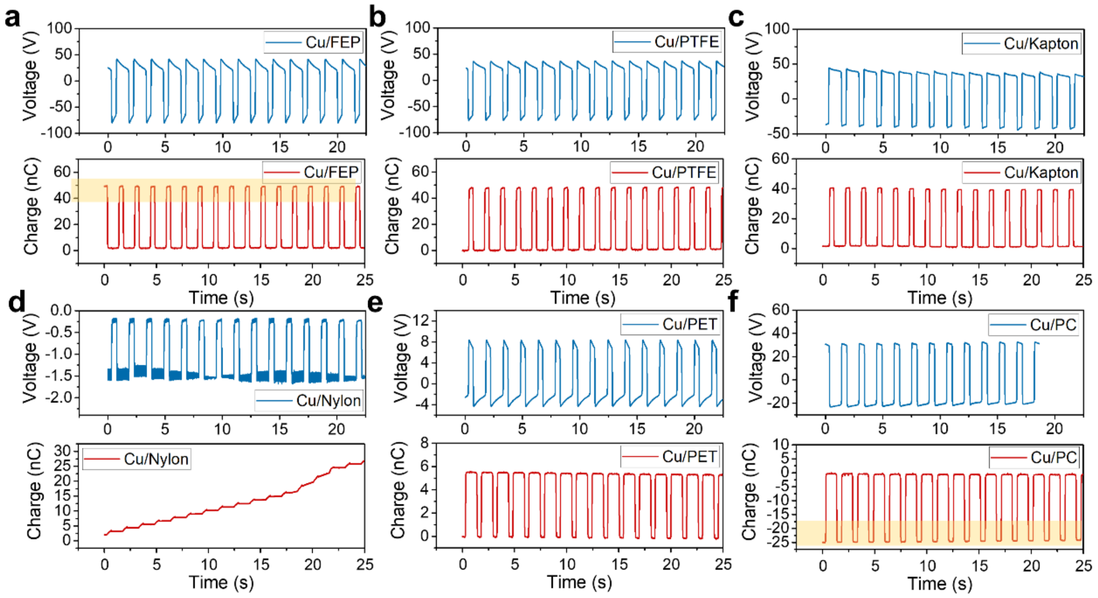

Supplementary Figure 21. V_{OC} and Q_{SC} of different Cu/polymer pairs. (a) Cu/FEP; (b) Cu/PTFE; (c) Cu/Kapton; (d) MG/Nylon; (e) Cu/PET; (f) Cu/PC.

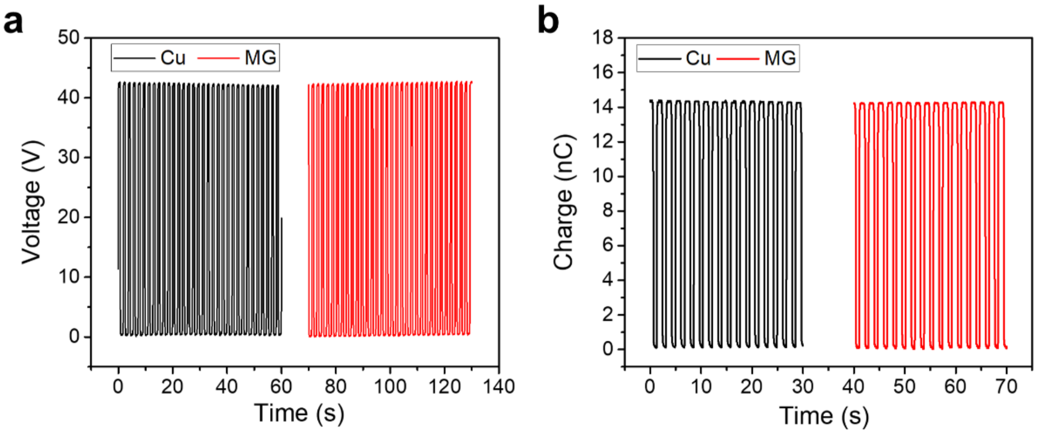

Supplementary Figure 22. V_{OC} and Q_{SC} of MG or Cu served as the induction metals. (a) Voltage; (b) Charge. No obvious difference can be noticed in the output, demonstrating that the output enhancement resulted from the MG directly contacting with polymers.

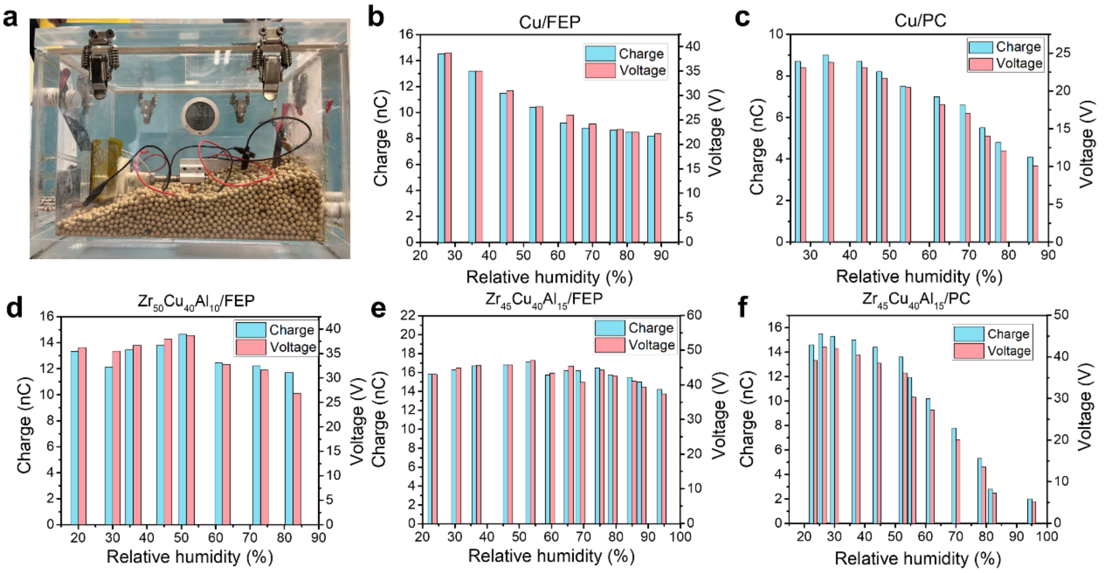

Supplementary Figure 23. V_{OC} and Q_{SC} of MG or Cu under different RH. (a) System configuration; (b) Cu/FEP; (c) Cu/PC; (d) $Zr_{50}Cu_{40}Al_{10}$/FEP; (e) $Zr_{45}Cu_{40}Al_{15}$/FEP (f)$Zr_{45}Cu_{40}Al_{15}$/PC.

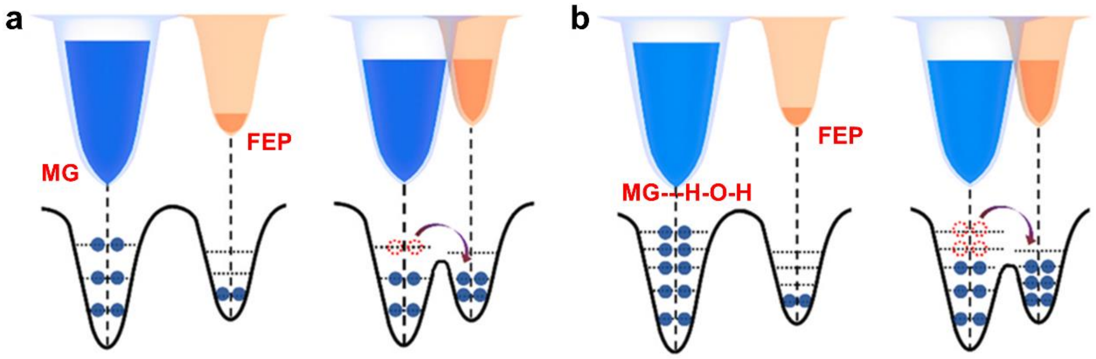

Supplementary Figure 24. Schematic diagrams of charge transfer mechanism of MG and MG with hydrogen bonding (MG—H-O-H) contacting with FEP.[1] Reproduced with permission. Copyright 2021, Elsevier Publishing Group.

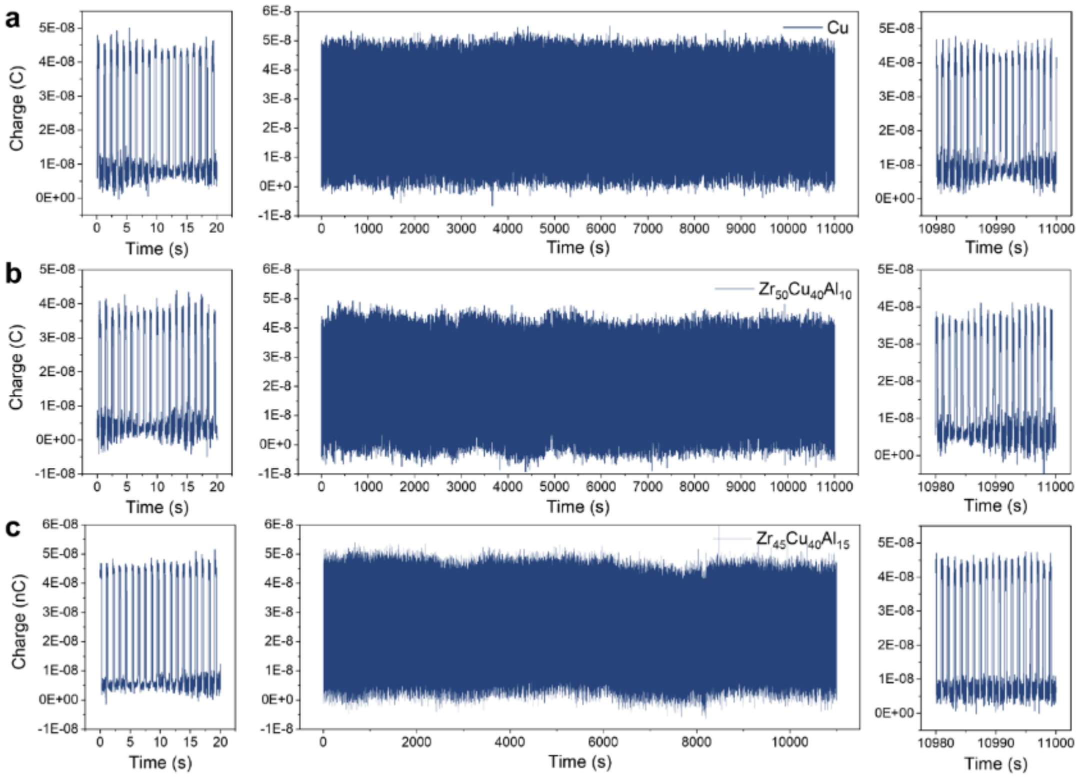

Supplementary Figure 25. Durability test. Long-term (11000s) charge measurement of (a) Cu/FEP; (b) $Zr_{45}Cu_{40}Al_{15}$/FEP and (c) $Zr_{45}Cu_{40}Al_{15}$/FEP pairs under soft substrate of 1 mm. Here the sample size is 5 mm × 25 mm and the excess displacement is 1 mm. The size of the FEP layer is 1 cm × 3.5 cm.

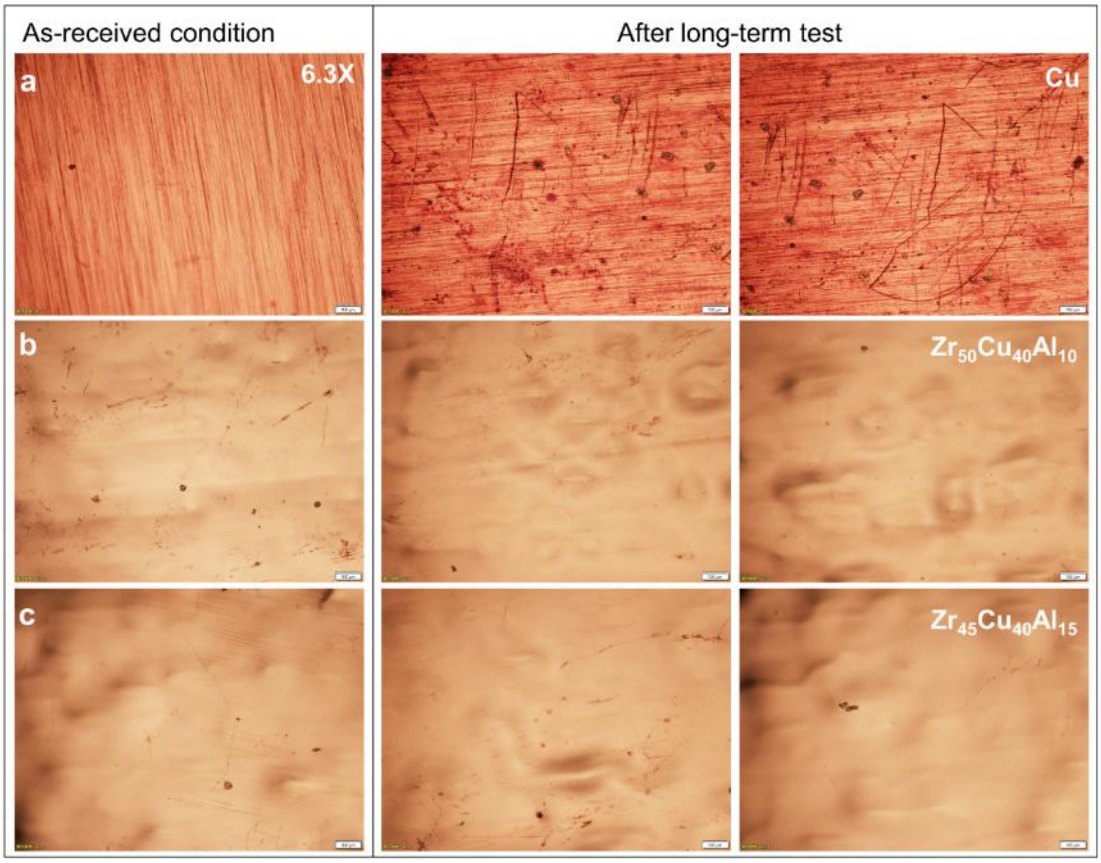

Supplementary Figure 26. Surface morphologies change before and after long-term durability test of (a) Cu, (b) $Zr_{50}Cu_{40}Al_{10}$ and (c) $Zr_{45}Cu_{40}Al_{15}$. Optical microscope results, scale bar of 100μm.

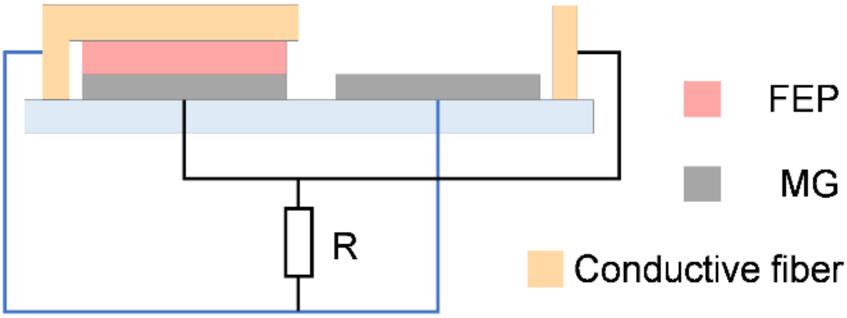

Supplementary Figure 27. Schematic structure of T-TENG.

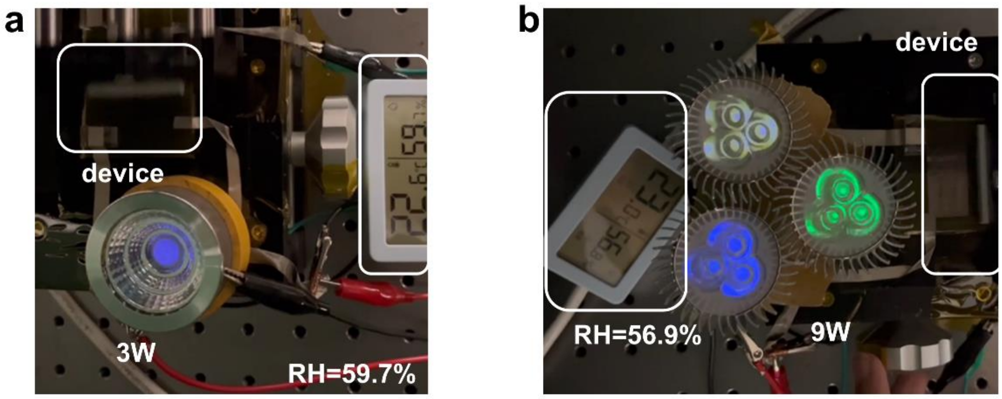

Supplementary Figure 28. System configuration for powering commercial LEDs.

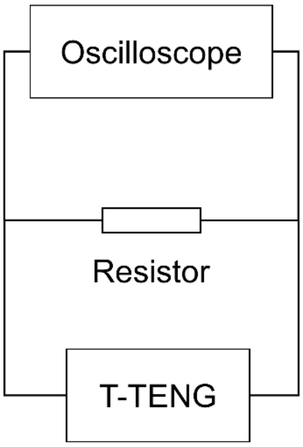

Supplementary Figure 29. Circuit for measuring the high frequency current.

Supplementary Table 1. Atom compositions of different samples from SEM-EDX results.

Samples	**Zr**	**Cu**	**Al**
$Zr_{45}Cu_{40}Al_{15}$	46.4%	37.8%	15.8%
$Zr_{50}Cu_{40}Al_{10}$	51.6%	38%	10.4%
$Zr_{45}Cu_{35}Al_{20}$	46.5%	33.1%	20.4%

Supplementary Table 2. Mechanical properties of different samples.

	**Cu**	**$Zr_{45}Cu_{40}Al_{15}$**	**$Zr_{45}Cu_{35}Al_{20}$**	**$Zr_{50}Cu_{40}Al_{10}$**
As (μm^2)	4.6851	0.301	0.35085	0.79832
H (GPa)	2.65	7.81	7.69	6.56
E(GPa)	142.57	107.16	110.33	91.99
K	0.20693	0.03918	0.04497	0.08728

Supplementary Table 3. Atomic density of different samples.

Samples	**Molecular weight (g/mol)**			**Density (g/cm^3)**	**Atomic density (×10^{22}/cm^3)**
$Zr_{50}Cu_{40}Al_{10}$	73.72855			6.910239404	5.83104048
$Zr_{45}Cu_{40}Al_{15}$	70.51643			6.521007979	5.75324807
$Zr_{45}Cu_{35}Al_{20}$	68.6882			6.214908483	5.62912973
Cu	63.546			8.351060367	8.17602216
Atomic weight (g/mol)	Zr	Cu	Al	Theoretical density of Cu:8.96g/cm^3; Measured density of ethyl alcohol: 0.79422g/cm^3 (theoretical: 0.7893g/cm^3 at 20℃).	
	91.224	63.546	26.9815		

Supplementary Table 4. Friction coefficient (dynamic) of different samples.

**Samples**	**Top surface**		**Bottom surface**	
	Friction Coefficient	Error	Friction Coefficient	Error
Zr5Cu4Al1	0.17898	0.04694	0.18448	0.04158

Zr4.5Cu3Al2	0.21277	0.02132	0.20886	0.05991
Zr4.5Cu4Al1.5	0.17982	0.03499	0.21313	0.06625
Cu	0.41825	0.05524		

Supplementary Table 5. Durability test of different samples.

Sample	Initial charge (nC)	Final charge (nC)	Charge reduction ΔQ (nC)	Decay ratio
Cu	33	30.1	2.9	8.788%
$Zr_{45}Cu_{40}Al_{15}$	37.4	34.5	2.9	7.754%
$Zr_{50}Cu_{40}Al_{10}$	30.2	27.2	3	9.934%

Supplementary Note 1. Calculation of the deformation involved in different devices.

As discussed in the manuscript, the MG samples were employed as the electrodes, as shown in the device photographs in Supplementary Figure 30c. In both contact-separation mode (Supplementary Figure 30a) and sliding mode (Supplementary Figure 30b) TENGs, the mechanical input with well-controlled force and displacement can hardly cause the severe plastic deformation that may induce fracture because the electrodes were fixed on the substrates through the Kapton tape serving as the "buffer" layer. With such the buffer layer providing additional elastic range, we didn't observe fractures in tests. For the sliding mode, the averaged maximized vertical load on the MG sample in Supplementary Figure 30b was around $\frac{20N}{30mm\times 25mm}\times 5mm\times 28mm = 3.73N$, and thus the maximized force of friction is around 0.75N, (friction coefficient of 0.2) which can be used to estimate the shear force. Thus, the elastic strain can be calculated by:

$$\varepsilon = \frac{F}{AE_r} \approx \frac{0.75N}{5mm\times 20\mu m\times 100GPa} = 0.75‱ \qquad (S.\ 1)$$

Similarly, for the CS mode TENG, the vertical load can be calculated by $F = \frac{m\Delta V}{\Delta t}$. Considering the maximized velocity of 0.1m/s, the impulse of the linear motor was around 0.3kg m/s. Here, the mass of the slider of motor is around 3kg. The time interval was selected from the charge output under d_e of 3.2 mm, as 0.2s (Supplementary Figure 30f). Thus, the maximized vertical load of CS mode TENG was around 1.5N. Then, during the relative sliding induced by the vertical load, the shear force was estimated at 0.3N (friction coefficient of 0.2) and the related strain was 0.3‱. Here, the friction of coefficient and the modulus were obtained by the indentation test, as indicated in Figure 1l and Supplementary Table 2 and Table 4. Therefore, the elastic strain range employed in this work was smaller than 1‱ for all modes, which is small enough to avoid the fractures during working.

Additionally, as we mentioned in the manuscript, among the three samples, only $Zr_{45}Cu_{35}Al_{20}$ is very brittle, which can be easily fractured by external forces. (Supplementary Figure 30d) Therefore, it was not utilized in further demonstrations in

this manuscript. The other two samples demonstrate better mechanical properties, where the as-spun ribbon can be bended or twined, as shown in Supplementary Figure 30e, which is stable enough for our tests, especially under such a low strain range.

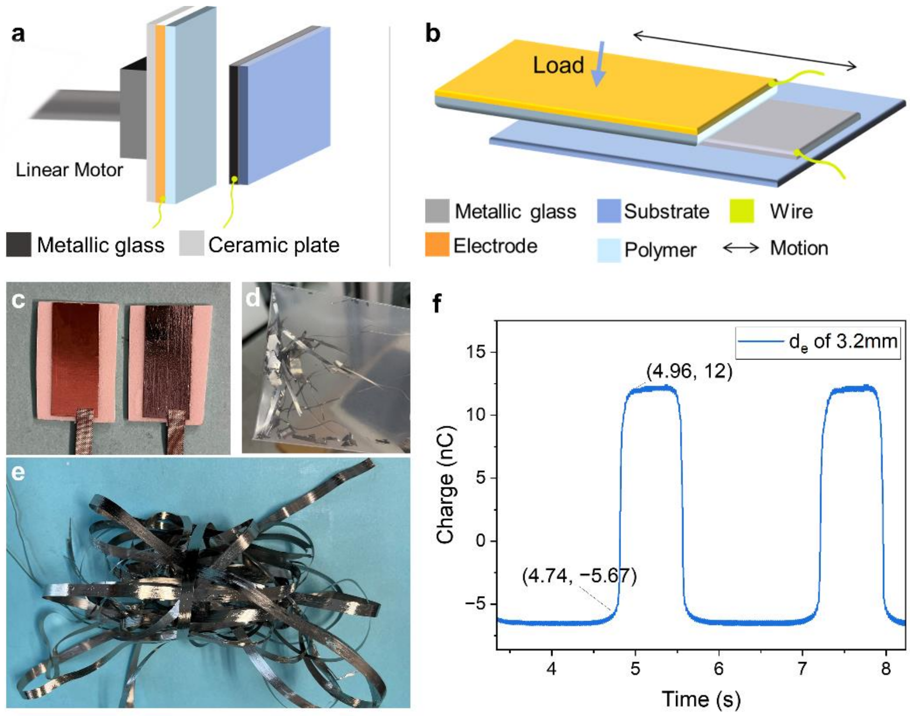

Supplementary Figure 30. Mechanical illustrations. (a) Setup of CS mode TENG. (b) Setup of LS mode TENG. (c) Photographs of electrodes. Photographs of (d) $Zr_{45}Cu_{35}Al_{20}$ and (e) $Zr_{45}Cu_{40}Al_{15}$. (f) Time interval of the CS mode TENG under d_e of 3.2mm. Here the electrode was $Zr_{45}Cu_{40}Al_{15}$.

To better understand the mechanical properties of the MG samples, we investigated the elastic strain limit through the nano-indentation measurement. The relationship between reduced modulus and elastic reduced modulus can be investigated through the following equation:[2]

$$\frac{1}{E_r} = \frac{1 - v^2}{E} + \frac{1 - v_i^2}{E_i} \qquad (S.2)$$

Here, E_r is reduced modulus; E is elastic modulus E_i is elastic modulus of diamond; v_i is poison's ratio of diamond; v is the poison's ratio, which can be assumed as 1/3.

After obtaining the elastic modulus, we simply assumed the yield strength as 1/3

of the hardness of materials. Then, the elastic strain limit ε can be calculated through the following equation:

$$\varepsilon = \frac{yiled\ strengh}{elastic\ modulus} \qquad (S.3)$$

The related parameters were obtained through the nano-indentation measurement, as depicted in Methods, and the final results of the elastic strain limit were summarized in the table below:

Supplementary Table 6. Elastic strain limit of different MG samples.

**Sample**	**E_r (Gpa)**	**v**	**v_i**	**E_i (GPa)**	**E (GPa)**	**Hardness (GPa)**	**Yield strength (GPa)**	**ε**
$Zr_{45}Cu_{40}Al_{15}$	107.16	1/3	0.07	1100	105.48	7.81	2.604	0.0247
$Zr_{50}Cu_{40}Al_{10}$	91.99	1/3	0.07	1100	89.20	6.56	2.186	0.0246
$Zr_{45}Cu_{35}Al_{20}$	110.33	1/3	0.07	1100	108.94	7.69	2.562	0.0236

It can be noticed from Supplementary Table 6 that the elastic strain limit of the three MG samples was around 2.36%~2.47%, which was much larger than the involved elastic strain in our experiment calculated previously, which is in ~‱ scale, further confirming the stability of the MG samples in our experiments.

Supplementary Note 2. Discussion of the different power output curve by T-TENG.

For TENG in most previous studies, the peak power of TENG usually increased first and then decreased with the resistance continuously increasing, resulting a maximized peak power appeared at a large matching impedance (larger than 10MΩ). However, by our design of transistor-like TENG in Figure 5e, the peak power decays with the increasing resistance. The different peak power output curve among our work and previously published works resulted from the integrated switches in our work. Generally, charge accumulation happens along with power output during the periodic motions in a common TENG in previous studies.[3] When a TENG directly connects to

a resistor, the voltage across the load will vary with the load, and a maximized peak power exists at the large impedance resistance. However, with the integrated switches on both sides in our design, the structure is under short-circuit condition at starting and ending of each working cycle, as shown in Supplementary Figure 27. During periodic motions of the transistor-like TENG, charge accumulates when the switch is off and then the whole charge releases instantaneously when the switch is on. Thus, when the resistance R increases, the peak power output decreases as $P_{\text{peak}} = V^2/R$, resulting a totally different characteristics compared with previous studies.

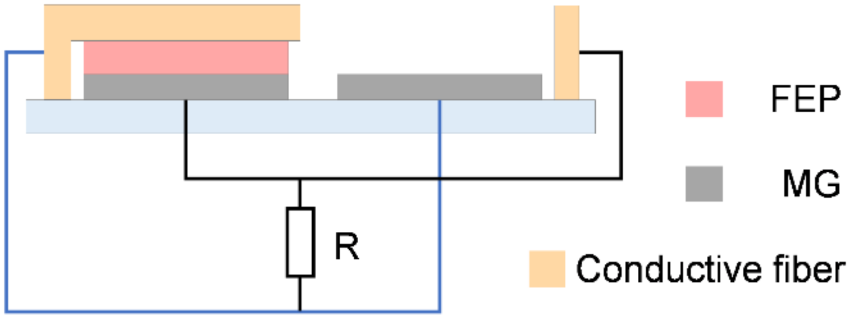

Supplementary Figure S27. **Schematic structure of T-TENG.**

To further confirm the output characteristics, the experiments were repeated, where the peak power decreased with the increasing resistance, as shown in Supplementary Figure 30. Here, a maximized peak power density around 8.34MW/m^2 was realized in the experiment at the RH around 68%, further confirming the output performance of the MG-based T-TENG. As the $P=V^2/R$, a slight voltage change may result in a significant charge in peak power, and thus the peak power density was changed. The decreasing in peak power density may result in the different slider as well as the higher RH, but the output characteristics are the same in the repeated experiments with well output performance.

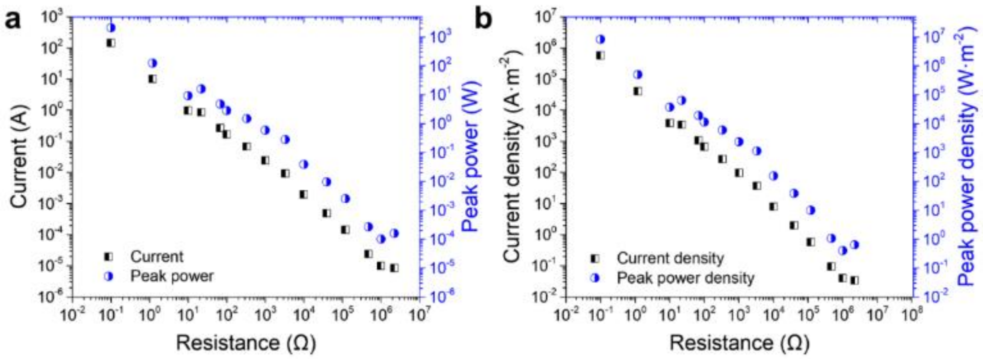

Supplementary Figure 31. Resistance impedance of T-TENG. Relative humidity around 68%.

References for supplementary information only:

1. Liu D, *et al.* Performance enhanced triboelectric nanogenerator by taking advantage of water in humid environments. *Nano Energy* **88**, (2021).

2. Bao YW, Wang W, Zhou YC. Investigation of the relationship between elastic modulus and hardness based on depth-sensing indentation measurements. *Acta Materialia* **52**, 5397-5404 (2004).

3. Niu S, Wang ZL. Theoretical systems of triboelectric nanogenerators. *Nano Energy* **14**, 161-192 (2015).
