# Supplementary material for: Metallic glass-based triboelectric nanogenerators
Source: Nat Commun. 2023 Feb 23;14:1023. doi: 10.1038/s41467-023-36675-x (PMC9950355; doi:10.1038/s41467-023-36675-x)
Supplement: Supplementary file 3 — Description of Additional Supplementary Files [file 41467_2023_36675_MOESM3_ESM.pdf]

Description of Additional Supplementary Files

File Name: Supplementary Movie 1
Description: 200 LEDs connected in series powered by the T-TENG.

File Name: Supplementary Movie 2
Description: LED of 3W powered by the T-TENG.

File Name: Supplementary Movie 3
Description: 3 3W-LEDs (9W in total) connected in series powered by the T-TENG in dark room.

File Name: Supplementary Movie 4
Description: 3 3W-LEDs (9W in total) connected in series powered by the T-TENG in bright room.

File Name: Supplementary Movie 5
Description: Demonstration of the small vertical load during measurement.
